# Doctors Dealing With COVID-19 in Pakistan: Experiences, Perceptions, Fear, and Responsibility

**DOI:** 10.3389/fpubh.2021.647543

**Published:** 2021-12-02

**Authors:** Inayat Ali, Salma Sadique, Shahbaz Ali

**Affiliations:** ^1^Department of Social and Cultural Anthropology, University of Vienna, Vienna, Austria; ^2^Community Health Sciences, Peoples University of Medical and Health Science Women, Nawabshah, Pakistan; ^3^Sindh Institute of Ophthalmology and Visual Sciences (SIOVS), Hyderabad, Pakistan

**Keywords:** COVID-19, pandemic, healthcare providers, anxiety, fear, Sindh, Pakistan, coping mechanism

## Abstract

This study aimed to describe the dealings of 20 biomedical doctors with coronavirus disease-19 (COVID-19) in the Sindh province of Pakistan. Focusing on physicians from three different hospitals, we describe their challenges, emotions, and views concerning the pandemic. Many regarded the virus from a biomedical standpoint. Yet some also perceived it as a “tool of a proxy war” and a “plot,” without giving agency to anyone for that “plot.” Furthermore, these care providers faced a great fear of infection and an even greater fear of transmitting the virus to their families and friends. A few also feared stigmatization as viral carriers. Whether they experienced fear or not, all of our physician interlocutors emphasized their sense of responsibility to “serve humanity,” yet some also expressed a strong belief in the inevitability of the will of Allah. Some were satisfied with the role of the government in containing the virus, while others expressed concerns and felt that the government should be doing much more. All expressed confidence in the use of personal protective equipment (PPE), viewing it as an effective buffer against viral contagion. We conclude with a call for further research especially ethnographic studies on dealings of physicians with COVID-19 across Pakistan as frontline care providers.

## Introduction

Amid a pandemic, frontline healthcare providers play pivotal roles and face critical impacts—social, psychological, and economic. Past pandemics have revealed such impacts, as healthcare providers dealing with people infected by severe acute respiratory syndrome (SARS) and the Middle East respiratory syndrome (MERS) were under extraordinary stress related to high risk of infection, stigmatization, understaffing, and uncertainty; consequently, comprehensive support for such providers was a high priority during these outbreaks and afterward (1–3). The primary sources of stress for providers have been shown to be fear of infection and of transmitting the disease to their families (2–4).

Current studies from across the world have pointed out severe impacts of the coronavirus pandemic—e.g., anxiety, trauma, and depressive symptoms—on healthcare providers, owing to increasing clinical responsibility, more chances to be infected, and possible family viral transmission (5–20). They have excessively experienced unpleasant emotions: fear, sadness, pain, uncertainty, and danger, along with intrusive memories, hyperarousal, and insomnia (20). This has happened due to multiple reasons such as extraordinary duties, overwhelmed resources, lack of thorough knowledge about the new virus, underdeveloped and frequently changing protocols, fear of auto-infection, and the risk of spreading the virus to their families and friends.

When we wrote this study in December 2020, there were a few studies, even from a public health perspective, on the impacts of the pandemic on Pakistani healthcare workers (HCWs). Now, while revising this study in August 2021, we have found that there are several recent studies conducted from that perspective; we present some of them here to help us make our points. Some studies have explored these impacts on healthcare providers in Pakistan, specifically. For instance, Sandesh et al. (21) found high levels of anxiety, stress, and depression among Pakistani healthcare providers related to coronavirus disease-19 (COVID-19). Khattak et al. (22) explored the psychological impacts that the fear of COVID-19 has exerted on nurses in the country. Similarly, Munawar and Choudhry (23) illustrated the stress experienced and coping mechanisms utilized by frontline emergency healthcare providers who have minimized their media exposure to avoid reading, hearing, and watching the news about COVID-19; many rely on religion to mitigate these challenging effects. Rana et al. (24) also indicated how healthcare providers have remained under physical and psychological pressure caused by fears of a high risk of infection, inadequate equipment for safety from contagion, isolation, exhaustion, and lack of contact with their families. Considering the importance of the Primary Health Providers' (PHPs) views, Hussain et al. studied the knowledge, attitudes, and practices toward COVID-19 in three tertiary care hospitals located in Peshawar, Khyber, Pakhtunkhwa (KPK) (25). While paying attention to the determinants of anxiety in physicians who worked in coronavirus wards or quarantine centers, Mahmood et al. found that there are substantial associations between “gender and anxiety” and identified specific needs of physicians, such as in relation to personal protective equipment (PPE), quarantine management, security and public support, and resource allocation (26). Moreover, Arshad et al. aimed to explore depression, anxiety, and stress (DAS) in HCWs and found that depression was significantly associated with the profession, and anxiety and stress were substantially associated with the age of HCWs: older ones wore more anxious and younger ones less (27).

Nonetheless, anthropological perspectives on healthcare providers to show the impacts of COVID-19, especially from the Sindh province of Pakistan, are still scarce. Therefore, this study aimed to explore and analyze the impacts of COVID-19 on biomedical doctors in Sindh and the coping mechanisms that they have adopted during the pandemic for dealing with people infected by this virus and with their own anxieties and fears.

## Methods and Materials

### Research Design, Participants, and Sampling

This study builds on ethnographic observations and fieldwork conducted during the COVID-19 pandemic, which was initially reported in Pakistan in February 2020. Employing qualitative research methods, we recruited biomedical doctors who were treating the patients suffering from COVID-19. These physicians worked in three COVID-19-designated hospitals in Sindh province: (1) Civil Hospital, (2) Peoples Hospital, and Taluqa [sub-district] Headquarter (THQ) Hospital. They participated in hour-long semi-structured in-depth interviews during August–September 2020. The convenience or purposive sample included 20 interlocutors serving in the frontline COVID-19 teams at these three hospitals. These physicians were approached by author Salma Sadique, who had access to them because she is working in a medical university.

### Data Collection

Using an interview guide, we focused our interviews with doctors on our key questions for them: (1) knowledge and perceptions around COVID-19; (2) social and psychological effects; (3) opinions about the dealings of the government with the pandemic; and (4) opinions about the end of the pandemic. Moreover, we carried out content and document analysis of gray literature, such as news reports and various surveys, mainly government reports, to contextualize the pandemic in Pakistan as background for understanding our interview results. Whenever possible, social scientists, mainly anthropologists, obtain their data in the native language due to several important factors, such as ethical considerations, valuing the local language, and collecting accurate data. Thus, all interviews were conducted in the Sindhi language, as this language was the mother tongue of these doctors. Salma Sadique conducted 18 interviews face-to-face and 2 *via* telephone. The composition of interlocutors included men, women, Muslims, Hindus, young adults, and elder adults (see Table 1). All interlocutors were informed about the project and asked to give their consent. Although the data generated from these 20 interviews cannot be generalized across the province, these proved enough for this study, as we reached saturation in terms of themes and information provided. We drew on Sandelowski's concept of “informational redundancy”: that when a researcher listens to similar views time and again, she/he reaches data saturation (28). The same stands true in our context; when, during the interviews, Sadique heard the same comments repetitively, she realized that no new data were emerging, only redundancies. Thus, she stopped data collection. Thereafter, we started analyzing the already collected data. Moreover, in ethnographic research, the sample size may be small, but the data collected are in-depth (29). Similarly, our research is anthropological and, thus, qualitative in nature. Instead of quantification, it aimed to arrive at an in-depth understanding of the physicians interviewed and the COVID-related problems they faced. Without claiming that the results are representative of the entire population of the province, we can say that our research does provide insight into the perceptions of interlocutors of COVID-19 and the impacts of the pandemic as they experienced them.

**Table 1:** Characteristics of interlocutors.

**Gender**	**Age**	**Religion**	**Qualification**	**Income per month in US$**	**Living area**	**Duty station**	**Ward**	**Position**
Male	45	Hinduism	MBBS/FCPS	2,800	Urban	DHQ	Isolation ward	Surgeon
Male	43	Islam	MBBS/FCPS	2,800	Urban	DHQ	Isolation ward	Surgeon
Male	43	Islam	MBBS/FCPS	2,800	Urban	DHQ	Isolation ward	Surgeon
Male	37	Islam	MBBS/MD	2,100	Urban	DHQ	Isolation ward	Surgeon
Male	43	Islam	MBBS/FCPS	2,800	Urban	DHQ	Isolation ward	Surgeon
Male	42	Hinduism	MBBS/FCPS	2,800	Urban	DHQ	Isolation ward	Surgeon
Male	45	Islam	MBBS/FCPS	2,800	Urban	DHQ	Isolation ward	Surgeon
Male	53	Islam	MBBS/FCPS	3,000	Rural	THQ	Isolation ward	Surgeon
Male	57	Islam	MBBS/FCPS	3,200	Rural	THQ	Isolation ward	Surgeon
Male	50	Islam	MBBS/FCPS	3,000	Rural	THQ	Isolation ward	Surgeon
Male	48	Islam	MBBS/FCPS	2,800	Rural	THQ	Isolation ward	Surgeon
Male	43	Islam	MBBS/MD	2,100	Rural	THQ	Isolation ward	Surgeon
Male	50	Islam	MBBS/FCPS	3,000	Urban	THQ	Isolation ward	Surgeon
Male	40	Islam	MBBS	2,000	Urban	DHQ	Isolation ward	Duty Doctor
Female	32	Islam	MBBS	2,000	Urban	DHQ	Isolation ward	Duty Doctor
Female	40	Islam	MBBS	2,000	Urban	DHQ	Isolation ward	Duty Doctor
Female	40	Islam	MBBS/MD	2,800	Urban	DHQ	Isolation ward	Duty Doctor
Female	37	Islam	MBBS	2,100	Urban	DHQ	Isolation ward	Duty Doctor
Female	42	Islam	MBBS/FCPS	2,800	Urban	DHQ	Isolation ward	Surgeon
Male	45	Islam	MBBS/FCPS	2,800	Urban	DHQ	Isolation ward	Surgeon

Herein, we also draw on our extensive previous ethnographic fieldwork in Pakistan, specifically in Sindh province—Inayat Ali (2005-present), Salma Sadique (2013-present), and Shahbaz Ali (2012-present)—in which we have focused on health and illness. It is important to mention that this study is part of a long project on COVID-19 that has been approved by Pakistan's National Bioethical Committee (reference no. 4-87/NBC-471-COVID-19-09/20/). The names of interlocutors have been anonymized to maintain confidentiality.

### Data Analysis

Although data analysis was ongoing from the first interview, the gathered data were subjected to content analysis. After transcribing the data into English, we continued reading and re-reading the data to gain familiarity with it and allowed for iteration. This transcription was followed by the data examination using thematic analysis and with the flexibility to extend, modify, and discard categories (30); this helped to identify the most salient themes. We then followed on by listing, summarizing, reviewing, and refining these various themes. Our analysis addressed the following questions: (1) How do doctors perceive COVID-19, and what practices did they perform to deal with it? (2) What kind of effects has the pandemic exerted on these frontline healthcare providers? (3) What are their perceptions of the government's policies and practices around dealing with the pandemic? The data obtained were eventually organized in terms of these questions and verbatim responses of interlocutors, which we present below. We grounded our categories in our data and theoretical framework (31) while agreeing with Dey [(30), p. 17], “There is no single set of categories waiting to be discovered. There are as many ways of “seeing” data as one can invent.”

## Background and Context

Although it has been mentioned by several studies [e.g., (32, 33)], in this section, we provide a brief overview of COVID-19 in Pakistan, and especially in Sindh province, to give our readers an idea of the overall situation with which our interlocutors are having to cope. Pakistan reported its first infection of COVID-19 in two men who returned from Iran on February 26, 2020. Although the government implemented several measures for containing the virus and “flattening the curve,” over time, infections increased. At the beginning of the pandemic, the Pakistani government suspended flights to and from several countries, such as China, Iran, Qatar, and Italy (34). In the absence of test kits in the country, the government sent specimens to China and the USA and, afterward, imported around 1,000 kits from China (34). On March 13, 2020, when the virus had infected only around 30 people, the government closed educational institutions and the border with Afghanistan and opened a quarantine camp for COVID-infected people at the Pak-Iran border. Later on, the country banned congregations, such as conferences and gatherings, both formal and informal. On March 17, 2020, on the basis of the information that 97% of patients have recovered, the Prime Minister of the country ruled out the option of countrywide lockdown (34). Nevertheless, the Sindh government implemented a lockdown in the province.

Afterward, the federal government did implement a countrywide lockdown, deployed security forces to enforce the preventive measures, such as forcing the entry of COVID+ people into quarantine centers, invoked Section 188 of the Pakistan Penal Code for any violations, monitored inter-provincial borders, closed the markets, created a Corona Relief Tiger Force to educate people about the critical consequences, distributed food items among daily wage laborers, and approved a PKR1.2 trillion economic relief package (34).

On May 9, 2020, the government lifted the countrywide lockdown due to its devastating effects on the economy and introduced and implemented a “smart” lockdown to keep only virus hotspots under lockdown. Under this smart lockdown, educational and training institutions, sporting, social and religious events, restaurants (except for take-away), marriage halls, cinemas, and business centers were closed.

When we first wrote this study in December 2020, the “smart” lockdown was still under operation, yet many people had long been and are still arranging gatherings and organizing marriage ceremonies. By December 30, 2020, Pakistan had reported COVID-19 infection in over 475,000 people, out of which around 10,000 had “officially” died. This very small number of deaths and cases out of a population of 22 billion seem a government fabrication to make it appear that they are doing an excellent job of coping with COVID-19 (35). Moreover, particular socio-cultural, economic, and political factors create a breeding ground for the virus to exert severe consequences in the country (32); we detail some of these below. As of August 2021, “smart” lockdown is still in place in some cities, such as Islamabad. There was great confusion and contestation going on between the political parties as to whether the cities should be locked down or not and between the federal government and provincial governments, as the Sindh government announced a province-wide lockdown in Sindh province, for which the federal government criticized the Sindh government. Nonetheless, later on in August 2021, the former government did implement a countrywide lockdown as the number of infections increased. By mid of August 2021, ~1,095,000 had contracted the virus and around 24,500 had died.

Views of the doctors, uncertainties, fears, and anxieties as described in this study should also be situated within the overall context of the healthcare system of Pakistan, which suffers from serious issues of untrained staff, lack of staff, such as lack of doctors, insufficient medicines and medical tools, overcrowding in health facilities, the rural-urban divide in access to the healthcare system, and corruption (36). For example, Pakistan has only one doctor per around 1,000 people; one dentist per around 9,450; and one hospital bed per over 1,600 people. There are around 1,300 public sector hospitals, 5,530 Basic Health Units (BHUs), 700 Rural Health Centers (RHCs), and 5,680 dispensaries. There is a significant difference between rural and urban areas in terms of access, affordability, and effectiveness of the healthcare system: This has been called the “rural-urban bias” (36).

## Results and Discussion

This section presents the study results, discussing them in relation to other studies conducted in Pakistan. Since healthcare providers are the ones who directly encounter COVID-19-positive people, it is highly pertinent to study and analyze their knowledge, attitudes, perceptions, and practices toward COVID-19 (25). As previously noted, some studies have found substantial associations between gender, age, and DAS in healthcare providers in Pakistan (26, 27). Likewise, we have focused on the same thematic area but from a different geographical region and from an anthropological perspective. Moreover, our study focuses specifically on biomedical doctors, whereas others have focused on healthcare providers in general or on specific types of providers that have not included doctors.

### Knowledge and Perceptions of COVID-19 Among Pakistani Physicians in Sindh Province

Since all interlocutors were medical doctors, they understood the fundamental biomedical characteristics of the virus: (1) COVID-19 is a viral infection caused by severe acute respiratory syndrome coronavirus 2 (SARS-CoV-2); (2) physical contact and droplets from sneezing and coughing are some of the causes of its spread; and (3) it causes respiratory tract infection. They also understood that the novel coronavirus was first reported in Wuhan city of China and that, thereafter, WHO had declared it a global pandemic.

Nonetheless, a few of them also related the pandemic to the conspiracy theories that have been circulating in Pakistan (32, 37, 38). One doctor confided, “COVID-19 is a tool of proxy war and the consequence of a lab experiment.” Another physician postulated, “I think this pandemic is planned, and I feel this will end once the purpose is being served.” Yet they gave no information on why they had these thoughts, what they meant by “proxy war,” or what “purpose” they felt was being served. When asked further about these issues, these doctors refused to speak more deeply about them, perhaps out of fear of government reprisal; though their names are not provided in this article.

### Social and Psychological Effects: Fears, Anxiety, and Habituation

All of our 20 physician interlocutors agreed that COVID-19 has impacted them and other healthcare providers socially, mentally, and economically, leading to massive stress and anxiety about becoming infected and infecting others. They are limited to contacts with only close family members; thus, they know that the ones they could infect are their nearest and dearest. The primary coping mechanisms they have developed include an attitude of optimism and carefully following standard operating procedures (SOPs) at every step while carrying out their hospital duties. One doctor stated, “I am maintaining SOPs, avoiding regular contact with anyone, and eating healthy foods for immunity.” Another doctor emphasized his faith in PPE, “I am unafraid of COVID-19. I respond positively to incoming patients by observing SOPs such as using gloves, masks, sanitizers, soaps, and other materials to protect myself. If you prevent yourself [from getting infected], you will protect others.” Another interlocutor agreed:

Many changes have occurred in my life since COVID-19 began—from maintaining physical distance, literally staying away from patients and family members with specific thoughts and anxieties that can I also infect my own family members. Although coping with these effects was not easy, practicing SOPs once again proved a relief in maintaining harmony between my conscience and duty.

Clearly, these physicians view PPE as an effective buffer against self-contagion and infecting others. Yet they still suffered from the physical distancing measures, they felt they had to employ. One doctor explained, “COVID-19 has affected me socially as I have to keep a distance from everyone.” Another doctor also discussed the social effects of the pandemic, “We cannot meet our families. Everything is different at home. To relieve stress, we are in touch with people through phone and video calling. Distancing from loved ones is quite challenging for me.”

Another doctor acknowledged that “In dealing with COVID-19 patients, I have some phobia. Otherwise, it is the best experience” to serve during these challenging times. Another explained how he has habituated to the realities of COVID:

While taking care of people infected with COVID-19, I have experienced growing fear and anxiety. On the first day, I was nervous about myself and my family. I was petrified to visit infected people, and my fear [of infection] increased continuously. However, now I have adapted to the unique environment of the hospital, and I can manage people infected by this virus with precautionary measures. The most challenging things were the psychology of infected people, as they were fearful. I have continually counseled them. It's been challenging times because no proper research is available, but only prophylactic treatment. Initially, I felt fear, but now I am used to handling the pandemic.

Another interlocutor also described his habituation:

At first, I was very much worried about the health of my family members. I was thinking to move to another place, but now I am used to it, which has relieved my social and mental disturbances.

Yet many of our interlocutors remained focused on their fears for their families and their distress over the lack of contact with family members:

It has challenged my life. I am anxious about how to protect myself, family members, and colleagues because I have seen those patients who are affected by COVID-19 in a critical situation.

I feel incredibly sad when my family feels fear from me, avoid touching me or my belongings. Every time I go [home], I need to clean or sanitize myself.

I am worried about my family and friends, thus, am trying to avoid close contact with them. I also follow the SOPs at home and workplace.

I am not even afraid of death but worried about loved ones who can be infected by me.

Being a healthcare provider working in COVID-19 wards causes significant stress, as I have been thinking of family. If I got infected, it might infect and affect my family.

I always feel anger and frustration due to my work as I am worried about the health of my loved ones.

I fear contracting the disease and I don't want to put my family and friends at risk, so I feel we all should take proper precautionary measures.

The main problem includes dealing with COVID-19 infected people and being exposed to the infection and being afraid of spreading it to my family members. As a result, I am substantially affected and physically distancing.

Although taking care of the patient is part of my job's responsibility, I am conscious of my loved ones due to my work's nature.

This doctor expressed the stigmatization that can occur to frontline workers:

At the start of the outbreak, I did not even allow myself to contact other people, especially my elder family members like grandparents, since they were vulnerable due to age. Most of my family members treated me as if I were the virus. After working in a COVID-19 ward, everyone avoids us [healthcare providers] as they think that we are a carrier of the virus.

In addition, this doctor described a new worry: people's resistance to following SOPs:

In the first phase, where cases were high and rapidly increasing in number, most community members were self-quarantining themselves, which ultimately relieved us and saved us from exposure to the virus. However, now it is very usual: nobody is taking precautions, even our management and so-called public health experts.

This last quotation indicates that since the coronavirus at that point had been around for several months, and now (August 2021) has been around for a year-and-a- half, Pakistanis are used to its presence and, probably due to the low death rate from the virus as reported by the government, are not taking it as seriously as they initially did. This may be a global problem—for example, in many cities in the United States, traffic levels are back to normal as people resume movement, more people are traveling on airplanes, and many are no longer wearing masks, despite the latest coronavirus wave of the Delta variant and the rapidly increasing number of infections in that country. According to the US citizen Robbie Davis-Floyd (personal communication, January 2021), people in the US are getting “COVID-ed out” and are “in denial” about the still-pressing need to take every precaution. The same seems true in Pakistan, and this situation represents a clear and present danger for all. As of August 6, 2021, around half of the US population had been fully vaccinated; the new, mostly Delta variant, infections are clustering in the US states with low vaccination rates and high vaccine resistance (Chamberlain 2021) https://www.reuters.com/world/us/half-us-population-fully-vaccinated-against-covid-19-cdc-2021-08-06/. Vaccines have arrived in Pakistan, and around 10% of the population has been vaccinated (https://leadpakistan.com.pk/news/covid-19-pakistans-10-pct-population-fully-vaccinated/, leaving a long way to go, which, as in the US, will be rendered more difficult by high levels of vaccine mistrust, especially amid the rural population. Meanwhile, a number of infected people are still growing in both countries and others—not to mention the recent viral mutations being experienced in the UK and Ireland, which may spread well and render the current vaccines ineffective.

### New Knowledge, Opportunity, Responsibility, and Guilt

Despite their anxiety and fear, some of our interlocutors considered the pandemic as an opportunity to gain new knowledge. One physician stated, “In contrast to its effects on healthcare professionals, this new disease has increased our existing knowledge, specifically on how to deal with a pandemic.” Similarly, another doctor stated, “Dealing with minor and major complications faced by patients is a bit challenging, but it feels great to be a part of it to learn new things.” Another doctor added, “It is quite a great experience. We are learning many new things. Since we had never dealt with a pandemic previously, we doctors were afraid of getting infected; thus, we needed to gain the first-hand experience to deal with C+ people.”

Our interlocutors often found themselves “between a rock and a hard place,” when they simultaneously invoked their responsibility to serve humanity and noted their guilt should they fail to do so. One doctor argued, “Although I am doing my job, sometimes I still get that feeling of guilt that I am not doing my duty to keep these people safe.” Four other doctors stated similar, yet differing, aspects of their feelings of both responsibility and guilt:

It is an inconvenient situation around the globe. Being a health professional, it's our responsibility to serve humanity. This is our duty to take better care of COVID-19 patients instead of succumbing to fear of getting infected.

As healthcare providers, our work is to face any critical situation. It's quite a challenging and a new experience, although the fear of being infected has become a part of our lives.

I feel proud to serve humanity, and I feel no fear while attending to patients with various diseases, including COVID-19. Nonetheless, I maintain the required SOPs, especially PPEs.

Saving the lives of humans is the responsibility of doctors. That is why I like this profession more because I serve humanity. I don't fear being infected. If it is God's will and in my destiny, then no one can turn it away.

This latter quote indexes the deep belief of Pakistanis in the cultural concept of *Qismat*, or “fate” — the notion that one's destiny is predetermined and inevitable. Nonetheless, Pakistanis, in general, do try to affect their destinies *via* prayer, the performance of religious rituals and, in the case of COVID, *via* the conscientious use of PPE—at least in urban areas, rural people living in small communities are far less likely to use PPE and far more likely to use ritual and prayer. Some rural people in Sindh province do not even believe that COVID-19 exists; they too consider it to be a “Western plot” or conspiracy (33).

### Fear of Syndemics

The “syndemics” — synergistic and epidemic—approach was introduced by Merrill Singer (39); it also combines the idea of “synergy” with “epidemic” and recognizes that diseases in a population are significantly related to sociocultural, economic, political, and ecological factors. In addition, syndemics entail the problematic interaction of two or more diseases adversely affected by such conditions. Thus, we employ this term to mean interactions between/among various infectious diseases that cause severe complications, such as death as due to such critical interactions; for example, some of our interlocutors had more anxiety related to their older age and preexisting comorbidities. As one doctor worried, “Yes, of course, my age is a problem, which makes me among the at-risk people to be infected and develop severe complications.” Another doctor was diabetic: “On my first day, I was a little afraid because I had diabetes, thus I might be infected. I was the first person to work at a COVID isolation ward at my hospital. Initially, PPE was unavailable.”

The syndemics of COVID-19 have already been studied, especially in the contexts of diabetes, hypertension, and cardiovascular diseases (35, 40, 41). Some studies have explored the strong relationships between diabetes, chronic kidney disease (CKD), and COVID-19 (40, 42). Ssentongo et al. (43) showed that people with several underlying conditions, such as cardiovascular disease, hypertension, diabetes, and CKD, are at considerable risk of death from COVID-19 infection compared to people without these comorbidities. For example, in their sample of around 800 people who died after contracting COVID-19, Chen et al. (44) found that 28% of those who died had CKD.

### Opinions of Doctors of Government Viral Containment Measures

Concerning the efforts of the government to slow the viral spread and supply healthcare providers with the necessary PPE, our interlocutors shared mixed responses. Some doctors were satisfied with the dealings of the government with the pandemic. They felt that the government did allocate sufficient funds and sufficient PPE. For instance, one doctor argued:

I feel that the government has strong prevention and control measures. That is why the pandemic will be controlled very soon. However, after all, we have a large population, and many people are not serious about the pandemic. Thus, the government's role is greatly challenged.

Yet other doctors were critical about the role of the government, arguing that its preventive measures were insufficient. For example, one doctor explained that “COVID-19 has disturbed me economically due to an increase in expenditures to buy sanitizers and masks.” Although the government has repeatedly stated that it will provide full PPE to healthcare practitioners, these words of the doctor reveal that it has not done so sufficiently, possibly due to the corruption that is chronic in the country, including in the healthcare system.

These physicians felt that the government should heighten its efforts to control the pandemic by, for example, providing healthcare facilities with maximum screening kits, as Pakistan lacks the large-scale screening measures that would enable it to better prevent viral spread. Moreover, these doctors want to see strict government implementation of preventive measures, such as physical distancing and eliminating handshaking.

### When the Pandemic May End: The Contrast Between Religious and Biomedical Beliefs

According to some of our interlocutors, this disease “will never go away but will live with us in the future. Only we can protect ourselves by observing COVID-19 SOPs.” In contrast, one doctor believes that “Only Allah can end this spread as soon as possible.” These two quotes are very intriguing for the collision of cultures and belief systems they represent: the strong Muslim cultural and religious belief in the power of Allah, and the belief common in a biomedical culture that individuals can protect themselves when they have sufficient information about how to do so.

**Figure 1:**
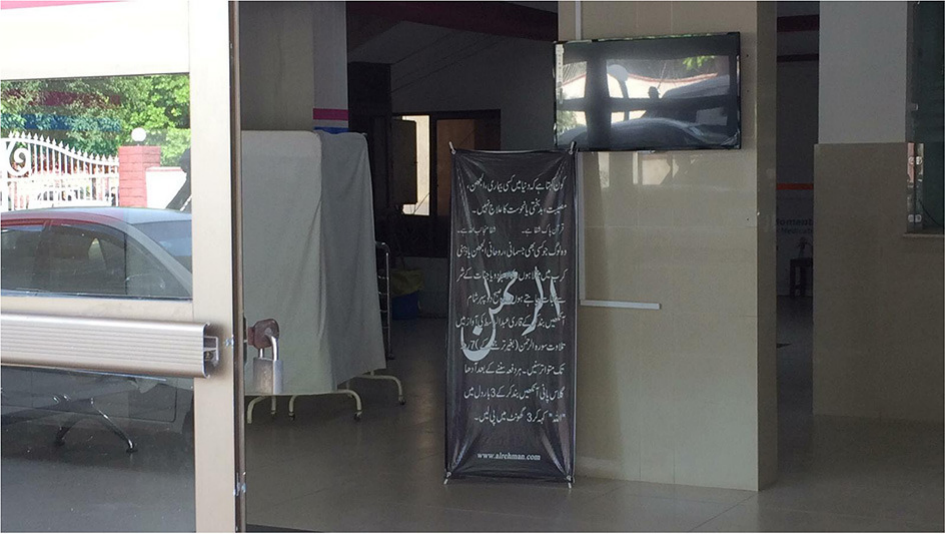
A banner at the entrance of COVID-19 ward containing the Urdu translation of a Quranic verse.

Although these doctors are trained in biomedical science, many determinately believe in a supernatural resolution.^1^ In every public and private hospital in Pakistan and even in clinics primarily for Hindus, there are either pamphlets hung on the walls or writings on the walls containing the Quranic verses that insist that no one can cure a disease but Allah. For instance, one verse reads, “And when I fall ill, it is He who restores me to health” (*Quran* 26:81) (see Figure 1). From this religious perspective, doctors can try as they might, but the primary protagonist is always Allah. This strong belief can work to absolve biomedical practitioners from guilt over a death they could not prevent and to give them faith that the death was the will of Allah. Yet if carried to its extreme, this belief would render biomedical efforts at healing pointless—and thus, in medical facilities, the biomedical belief that something can be done must prevail—despite those inscriptions on the walls.

Most laypeople in Pakistan also hold this religious belief, and therefore many consider the pandemic to be a “supernatural test” of their faith; thus, they have focused on performing religious rituals and reciting prayers and Quranic verses more than they have on taking preventive measures. After all, if it is Allah who determines your fate, would you not be better served by praying to Him? Even the Pakistani government arranged a communal, nationwide, televised prayer at the beginning of the pandemic (45).

As to its possible end, about which another doctor implored, “I just hope it ends soon as it's affecting thousands of people globally,” some of our interlocutors suggested that once the pandemic winds down, all preventive measures at home and work should continue to be practiced to avoid the spread of other infectious diseases, such as colds, the flu, the measles, and polio—of which Pakistan continues to have sporadic outbreaks due to lack of vaccination coverage (46). Moreover, other vaccine-preventable diseases, such as maternal tetanus, also still occur in the country for the same reason (47).

## Study Limitations and Strengths

This study has several strengths and limitations. Its greatest strength is that it is the first anthropological study from Pakistan to address the effects of COVID-19 on physicians as frontline healthcare providers. No other anthropological study has been conducted from that standpoint. There are studies from public health/epidemiological perspectives and from other geographical areas of Pakistan focusing on the knowledge, perceptions, and practices of other types of healthcare providers who have dealt with COVID-19-positive people. Yet, studies from Sindh province are scant. The limitation of this study lies in its small sample size of only 20 interviews with doctors in Sindh province; its results could be strengthened with a higher sample size and by the inclusion of doctors from other regions of the country. In addition, this study involved only biomedical doctors, whereas other studies have focused on other healthcare providers. Therefore, we suggest further research across the country. Regarding the sample size, we also would like to re-emphasize that, for the most parts, ethnographic studies do tend to engage with a smaller sample size, as their main strength lies in their detailed qualitative accounts. As far as we know, prior to our study, there is not a single study that has also focused on what biomedical doctors think about governmental measures and how they perceive the potential end of the pandemic.

## Conclusions

This study of biomedical physicians from the Sindh province of Pakistan has shown that while all interlocutors perceived the virus from a biomedical perspective, some considered it as a “proxy war's tool” and “the consequence of a lab experiment,” or a “plot,” without mentioning its agency or purpose. All believed in biomedical treatment, while some also believed that living or dying from COVID-19 was up to Allah. We have shown how COVID-19 has affected our interlocutors socially, psychologically, and economically. Socially, they had to be overcautious to maintain physical distancing, such as from their family members. Psychologically, they suffered stigmatization as suspected viral carriers and also suffered from lack of physical contact with family members—or having contact with them yet constantly being afraid of infecting them. They suffered as well from anxiety and fear of being infected or developing severe symptoms due to the “syndemics effect” that we described. Economically, a few interlocutors indicated an increase in their expenditures to buy PPE, which they believed should have been supplied freely by the government, as had been promised. Some were satisfied with the dealings of the government with the pandemic, whereas others showed great dissatisfaction and felt that much more should be done. Overall, these doctors keenly felt their responsibilities as frontline care providers and experienced guilt when they felt that they were not “doing enough.” Precisely because these physicians are essential frontline care providers in this pandemic, we believe in the importance of capturing and presenting their voices, their feelings, and their experiences.

## Data Availability Statement

The datasets presented in this article are not readily available because due to ethical reasons, we cannot share the dataset. Nonetheless, our paper contains interlocutors' responses in the original at several places. Requests to access the datasets should be directed to inayat_qau@yahoo.com.

## Ethics Statement

The studies involving human participants were reviewed and approved by Pakistan's National Bioethical Committee (reference No.4-87/NBC-471-COVID-19-09/20/). Informed consent for participation was not required for this study in accordance with the national legislation and the institutional requirements.

## Author Contributions

IA did conceptualization, wrote the first draft, contributed to analysis, revision, and validation. SS did data collection, wrote the first draft, revision, analysis, and validation. SA did revision, analysis, and validation. All authors contributed to the article and approved the submitted version.

## Funding

IA acknowledges the Higher Education Commission (HEC) of Pakistan's grant (PD/OSS-II/Batch-IV/Austria/2012/9903), which supported the Ph.D., work that has significantly informed this article.

## Conflict of Interest

The authors declare that the research was conducted in the absence of any commercial or financial relationships that could be construed as a potential conflict of interest.

## Publisher's Note

All claims expressed in this article are solely those of the authors and do not necessarily represent those of their affiliated organizations, or those of the publisher, the editors and the reviewers. Any product that may be evaluated in this article, or claim that may be made by its manufacturer, is not guaranteed or endorsed by the publisher.

## Footnotes

^1^Although unrelated to our actual interlocutors, we have seen a doctor in Sindh province who has clearly displayed a notice at the entrance to his facility stating that people of one specific Islamic sect are not allowed to enter his hospital. He will not treat them, as he believes that the Holy Prophet Muhammad would not want him to do so, due to their “erroneous” beliefs. His action reveals a different contestation, in which power, politics, religion, and biomedicine are intricately entangled. Moreover, his action belies those beliefs of our interlocutors that they should serve all of humanity. Undoubtedly, most of them do prioritize humanity. However, the smaller number of doctors who value religion over science and biomedicine are critical indications of the religious extremism extant in the country—extremism that is rooted in history and (geo-)politics.
